# Multiple roles of haem in cystathionine β-synthase activity: implications for hemin and other therapies of acute hepatic porphyria

**DOI:** 10.1042/BSR20210935

**Published:** 2021-07-22

**Authors:** Abdulla A.-B. Badawy

**Affiliations:** Formerly School of Health Sciences, Cardiff Metropolitan University, Western Avenue, Cardiff CF5 2YB, Wales, U.K.

**Keywords:** 3-Hydroxykynurenine, Acute intermittent porphyria, Carbon monoxide, Kynurenine pathway, Pyridoxal 5′-phosphate, Xanthurenic acid

## Abstract

The role of haem in the activity of cystathionine β-synthase (CBS) is reviewed and a hypothesis postulating multiple effects of haem on enzyme activity under conditions of haem excess or deficiency is proposed, with implications for some therapies of acute hepatic porphyrias. CBS utilises both haem and pyridoxal 5′-phosphate (PLP) as cofactors. Although haem does not participate directly in the catalytic process, it is vital for PLP binding to the enzyme and potentially also for CBS stability. Haem deficiency can therefore undermine CBS activity by impairing PLP binding and facilitating CBS degradation. Excess haem can also impair CBS activity by inhibiting it via CO resulting from haem induction of haem oxygenase 1 (HO 1), and by induction of a functional vitamin B_6_ deficiency following activation of hepatic tryptophan 2,3-dioxygenase (TDO) and subsequent utilisation of PLP by enhanced kynurenine aminotransferase (KAT) and kynureninase (Kynase) activities. CBS inhibition results in accumulation of the cardiovascular risk factor homocysteine (Hcy) and evidence is emerging for plasma Hcy elevation in patients with acute hepatic porphyrias. Decreased CBS activity may also induce a proinflammatory state, inhibit expression of haem oxygenase and activate the extrahepatic kynurenine pathway (KP) thereby further contributing to the Hcy elevation. The hypothesis predicts likely changes in CBS activity and plasma Hcy levels in untreated hepatic porphyria patients and in those receiving hemin or certain gene-based therapies. In the present review, these aspects are discussed, means of testing the hypothesis in preclinical experimental settings and porphyric patients are suggested and potential nutritional and other therapies are proposed.

## Introduction

The present review discusses the role of haem in control of activity of cystathionine β-synthase (CBS, EC: 4.2.1.22) and advances a hypothesis postulating that this control is exerted by the haem cofactor at multiple levels. This hypothesis was prompted by the reported [1,2] increase in the plasma concentration of the cardiovascular risk factor [3–6] homocysteine (Hcy) in patients with acute intermittent porphyria (AIP), which was attributed to inhibition of CBS activity by depletion of its other cofactor pyridoxal 5′-phosphate (PLP). Further evidence has very recently emerged for a greater plasma Hcy elevation in some patients undergoing a specific gene therapy (see below). Despite its indirect involvement in the CBS catalytic reaction, haem plays multiple roles in control of the enzyme and in the PLP depletion. These aspects will be examined here in detail. In the following text, brief descriptions of the haem-biosynthetic pathway, the hepatic porphyrias and Hcy metabolism will be followed by an account of the multiple roles of haem in control of CBS activity. Potential mechanisms of induction of PLP deficiency by haem in AIP will then be discussed with special emphasis on the role of PLP-dependent enzymes of the kynurenine (Kyn) pathway (KP) of tryptophan (Trp) degradation and their use as markers of the functional B_6_ status. Competition for PLP by the relevant enzymes of haem, Hcy and Trp metabolism will be discussed. Elevation of plasma Hcy levels by CBS inhibition in AIP will then be considered in relation to the potential harmful effects of this amino acid and the impact of certain AIP therapies. Next, the effects of haem will be summarised in a hypothesis predicting likely changes in CBS activity and plasma [Hcy] under basal conditions and following some AIP therapies. Finally, means of testing the hypothesis, and nutrition-based and other therapies countering the Hcy elevation and/or effects will be proposed.

## AIP

**Figure 1:**
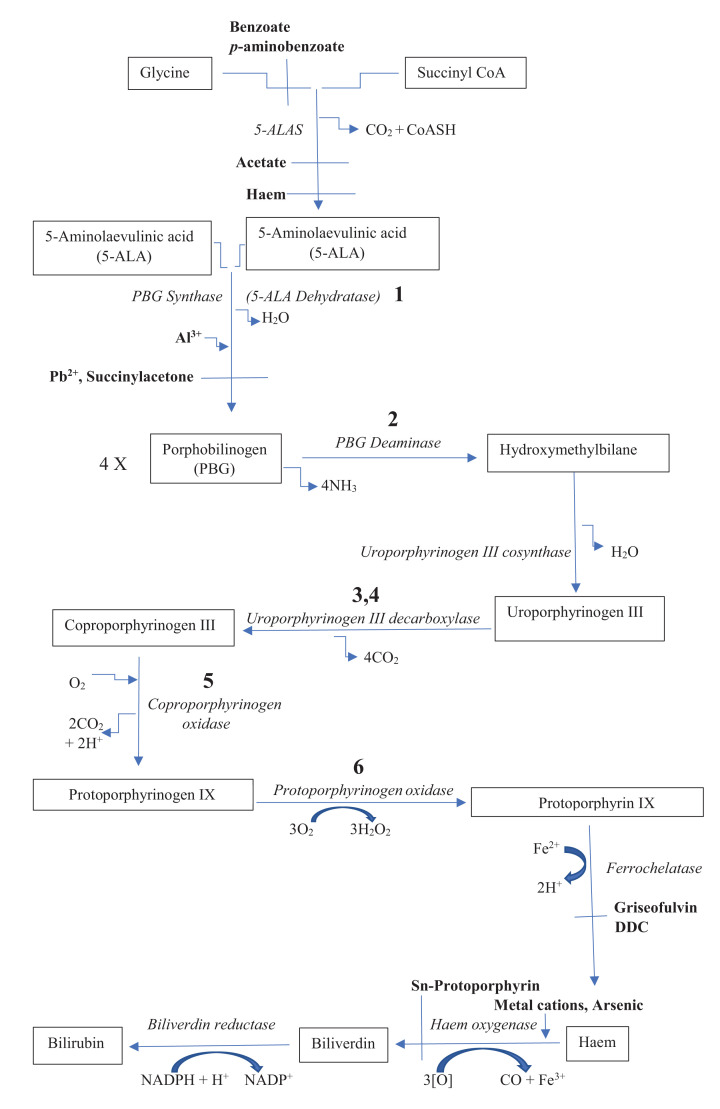
The haem-biosynthetic and degradative pathways Reproduced here from Figure 1 in [7] [Badawy, A.A.-B. (2019) Hypothesis: Metabolic targeting of 5-aminolaevulinate synthase by tryptophan and inhibitors of heme utilisation by tryptophan 2,3-dioxygenase as potential therapies of acute hepatic porphyrias. *Med. Hypotheses***131**, 10934 https://doi.org/10.1016/j.mehy.2019.109314]. Enzymes are in italic and inhibitors or stimulators are in bold letters. The bold numbers denote the enzyme defect in the following hepatic porphyrias: 1 (5-ALA dehydratase porphyria); 2 (AIP); 3 (porphyria cutanea tarda); 4 (hepato-erythropoietic porphyria); 5 (hereditary coproporphyria); 6 (variegate porphyria). Abbreviation: DDC, 3,5-diethoxycarbonyl-1,4-dihydrocollidine.

AIP is caused by a partial defect in the gene encoding one of the enzymes of the haem-biosynthetic pathway, porphobilinogen (PBG) deaminase (PBGD), the *hydroxymethylbilane* gene (Figure 1) [7] and is associated with acute attacks causing abdominal pain and neurological disorders that are precipitated by various drugs and chemicals, certain hormones and fasting [7]. It is thought that these symptoms are caused by the accumulated haem precursor 5-aminolaevulinic acid (5-ALA) due to the PBGD deficiency [7,8]. Attacks occur upon induction of 5-ALA synthase 1 (5-ALAS 1), the rate-limiting enzyme of the pathway, when hepatic haem is depleted, as haem exerts feedback control over the pathway at the 5-ALAS 1 step via a small regulatory pool in the hepatic cytosol, estimated to be ∼10^−7^ M [7,9] and used apparently exclusively by the minor cytosolic haemoprotein tryptophan (Trp) 2,3-dioxygenase (TDO, formerly Trp pyrrolase) (see the detailed discussion in [7]). Attacks are treated by intravenous glucose or hemin (haematin) [10], which act by blocking 5-ALAS 1 induction [7]. More recently, gene therapy has been introduced in various forms: 5-ALAS 1 gene silencing by Givosiran with proven efficacy [11,12], a liver-directed recombinant adeno-associated vector expressing PBGD, with proven safety, but not efficacy in reversing the increased urinary excretion of 5-ALA and PBG at the dosage used [13,14], and a bioengineered PBGD variant shown in experimental models to improve the therapeutic index of the original vector [15]. A preclinical study of intravenous human PBGD mRNA encapsulated in lipid nanoparticles also showed promising results in mouse and non-human primate models [16].

## Hcy metabolism and changes in AIP

**Figure 2:**
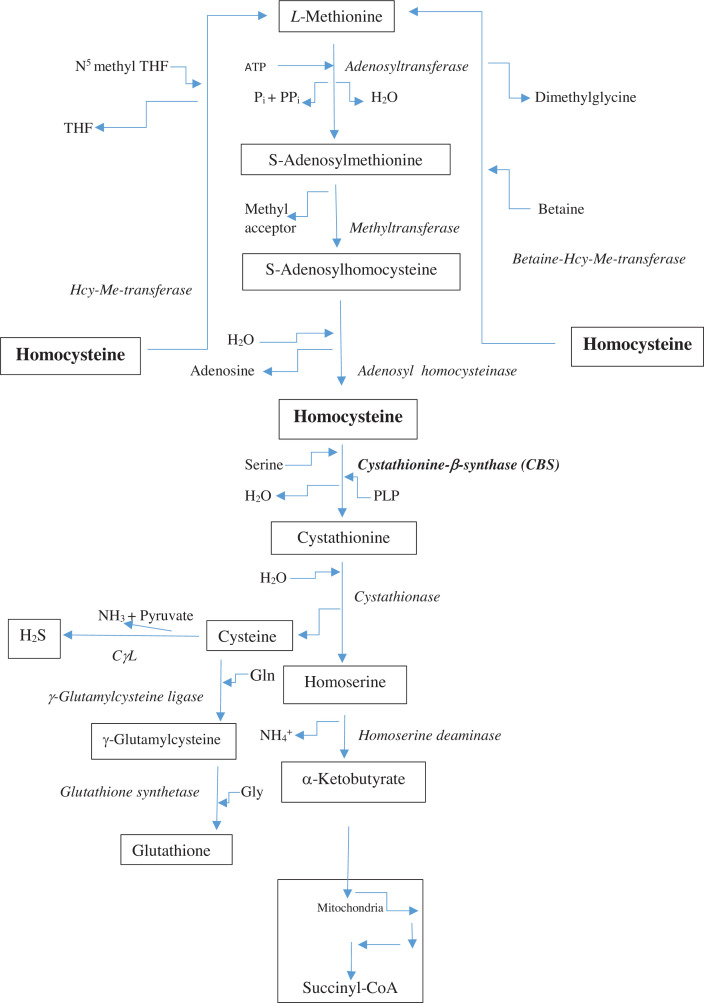
Hcy biosynthesis and degradation Abbreviations: ATP, adenosine triphosphate; CγL, cystathionine γ-lyase; Gln, glutamine; Gly, glycine; Me, methyl; P_i_, inorganic phosphate; PP_i_, inorganic pyrophosphate; THF, tetrahydrofolate.

Hcy synthesis and degradation are illustrated in Figure 2. Hcy is a product of methionine (Met) metabolism and its levels can be increased by excessive intake of Met-rich animal proteins [17]. Hcy is also converted into Met via two processes, the Met salvage pathway and methylation by betaine. The first can be impaired by a nutritional deficiency of folate or a genetic defect in the N^5^,N^10^-methylene-tetrahydrofolate reductase (*MTHFR*) gene, as in homocystinuria [5,6], both resulting in Hcy accumulation. As well as folate, vitamin B_12_ is also important, as it is required for the transfer of the CH_3_ group, such that, in B_12_ deficiency, methyl groups remain trapped in N^5^-methyl THF [18].

As Hcy is metabolised to cystathionine by PLP-dependent CBS, vitamin B_6_ is therefore the third B vitamin required in Hcy metabolism. Plasma [Hcy] can be increased by a nutritional or a functional B_6_ deficiency. The latter can be induced by drugs that bind and thus inactivate PLP, such as hydrazine compounds [19] or by increased PLP consumption upon activation of PLP-dependent enzymes, e.g. aminotransferases. Cystathionine is the precursor of cysteine through the catalytic action of cystathionase. The other product of the cystathionase reaction, homoserine, is deaminated to α-ketobutyrate, which is converted in mitochondria into the 5-ALAS substrate succinyl CoA through a four-step process involving branched-chain amino acid and odd-chain fatty acid metabolism [18]. Figure 2 also shows the production of H_2_S from cysteine by the catalytic action of cystathionine γ-lyase (CγL) and also production of glutathione from cysteine by successive incorporation of Gln and Gly.

In the two studies in AIP patients [1,2], plasma [Hcy] was elevated in ∼60% of small samples of patients (*n*=24–46), with no observed changes in serum folate or B_12_, but with decreased levels of PLP. [Hcy] was higher in symptomatic and biochemically characterised (by raised urinary 5-ALA and PBG levels), compared with asymptomatic, patients and was more pronounced in patients receiving haem therapy [2], though the earlier study [1] reported lower [Hcy] after haem therapy.

## The multiple roles of haem in CBS activity

Haem appears to exert a range of direct and indirect effects on CBS activity under normal physiological conditions and when haem levels are either decreased or increased. These effects can impact plasma Hcy levels in patients with acute hepatic porphyrias, both under basal conditions and during certain therapies.

### Normal and decreased haem availability

CBS utilises both haem and PLP as cofactors. However, haem does not participate in the CBS-catalysed conversion of Hcy into cystathionine, whereas PLP does. Haem is postulated to assume a regulatory role, since CBS activity is inhibited by CO or NO, both of which bind to haem [20]. With CO binding, a PLP shift occurs from the reactive ketoenamine, whose imine C=N group facilitates attack by the nucleophilic serine, to the inactive enolimine form [20]. CO is a product of the haem oxygenase 1 (HO 1) reaction and its physiological concentration is in the range of 3–30 µM. As affinity of CO for CBS is in the physiologically relevant µM range, it has been suggested that endogenous CO can inhibit CBS activity (see [20] for details and references). It follows therefore that HO 1 induction by haem (see below) is almost certain to impair CBS activity significantly. CBS activity is also influenced by the redox state of haem, with the Fe^2+^ state lowering enzyme activity [21]. Haem also stabilises CBS variants against proteolysis [22]. Thus, normal haem levels are essential for CBS activity. It follows therefore that a decrease in haem levels can undermine such activity leading to elevation of plasma Hcy. The haem status in AIP patients may therefore determine plasma Hcy levels, with normal levels associated with normal haem synthesis and raised levels occurring when haem synthesis is impaired by pharmacological, genetic or nutritional factors, e.g. by glucose therapy, inhibitors of haem-biosynthetic enzymes, 5-ALAS 1 gene silencing, HO 1 inducers, and unbalanced or malnutrition.

### Increased haem availability

CBS activity can also be impaired in the presence of raised haem levels, e.g. during haem therapy. It is noteworthy that raised plasma Hcy is pronounced in recurrent AIP patients receiving repeated hemin therapy [2]. Excess haem can inhibit CBS activity by two mechanisms. The first involves CO [20] produced from the haem oxygenase reaction following induction of HO 1 by haem. Induction of HO 1 mRNA expression, protein levels and/or activity by haem arginate has been demonstrated in rats, dogs and humans [23–27]. The second mechanism involves PLP depletion following its consumption by kynurenine aminotransferase (KAT) and kynureninase (kynase) following activation of liver TDO by haem (see further below).

The time after haem treatment is likely to be an important determinant of the plasma Hcy elevation due to CBS inhibition. A time-course study in rats [23] showed that HO 1 induction by haem arginate is highest (approx. five-fold) at 5–7 h and remains pronounced (approx. three-fold) at 24 h after the last of four daily injections of 15 mg/kg body weight each, with comparable levels of induction by hemin and haem arginate. At 24 h, loss of haem is apparent from the decreased levels of cytochrome *P*-450 and impaired metabolism of arachidonic acid [23] and several drugs [24]. Induction of HO 1 protein and activity in humans was still pronounced at 48 h after intravenous administration of haem–albumin [25] or haem arginate [26]. Plasma haem levels following intravenous haem administration are also elevated at 48 h. With haem arginate, plasma [haem] is increased from near zero at 0 h to ∼20 µM at 48 h [27]. With multiple dosing of haem arginate, the plasma T_1/2_ of haem rises from 11 h after the first dose to ∼18 h after the fourth dose [28]. 5-ALAS 1 activity was measured in only one study [24] in rats and dogs 24 h after a 30-day daily treatment with haem arginate. 5-ALAS 1 activity was unaltered in rats, but increased in dogs. From this account, it is likely that the time-dependent changes in haem levels and HO 1 activity following exogenous haem administration will determine the effects of haem on 5-ALAS 1, CBS and TDO activities and thus determine changes in plasma Hcy. The effects of haem on TDO activity have also been investigated in rats [29]. Acute administration of hemin or 5-ALA induces a strong saturation of the apoenzyme with haem with a maximum activation at 6 h and a return to basal levels by ∼16 h. Thus increased production of Kyn and the transamination products kynurenic acid (KA) and xanthurenic acid (XA) leading to PLP deficiency in AIP patients (see further below) can be expected to occur over the first 24 h after a single dose of haem or for prolonged periods with multiple dosing. In this latter situation, the PLP depletion should be stronger than after single dosing. PLP depletion in AIP will be considered in the following section.

## The vitamin B nutritional status in AIP

### General nutrition

Most studies of the nutritional status of AIP patients centred round the need for intake of a balanced diet, suggesting an imbalanced nutritional status in this population. A controlled study [30] of a small number of AIP patients (*n*=16) reported decreased carbohydrate intake, increased lipid intake and greatly increased protein intake, with inadequate Zn, folate and tocopherol intakes. The very high protein intake can promote Hcy formation from Met-rich proteins [17]. The low folate intake can undermine the conversion of Hcy into Met, further contributing to the Hcy elevation [5,31,32]. The low Zn intake can undermine the Zn-dependent 5-ALA dehydratase (PBG synthase) activity (Figure 1) [33,34], thereby possibly contributing further to the 5-ALA accumulation [8]. For example, the loss of Zn during the first 24 h of serum-free culturing of non-proliferating rat hepatocytes is associated with a 95% loss of 5-ALA dehydratase activity and over 85% loss of its immunoreactive protein [33], and rats maintained on Zn-deficient diets exhibit decreased enzyme activity that responds only mildly to Zn supplementation [34], a finding suggested by the authors to be due to an effect of Zn deficiency at the level of enzyme synthesis. Thus, this nutritionally unbalanced dietary intake by porphyria patients can contribute to fluctuations in, and elevation of, plasma [Hcy], even in the absence of pharmacological and other therapies, and, in fact, plasma [Hcy] is generally mildly elevated in porphyric patients in the absence of therapeutic interventions (see below) .

### Plasma CBS-related vitamin B levels

**Figure 3:**
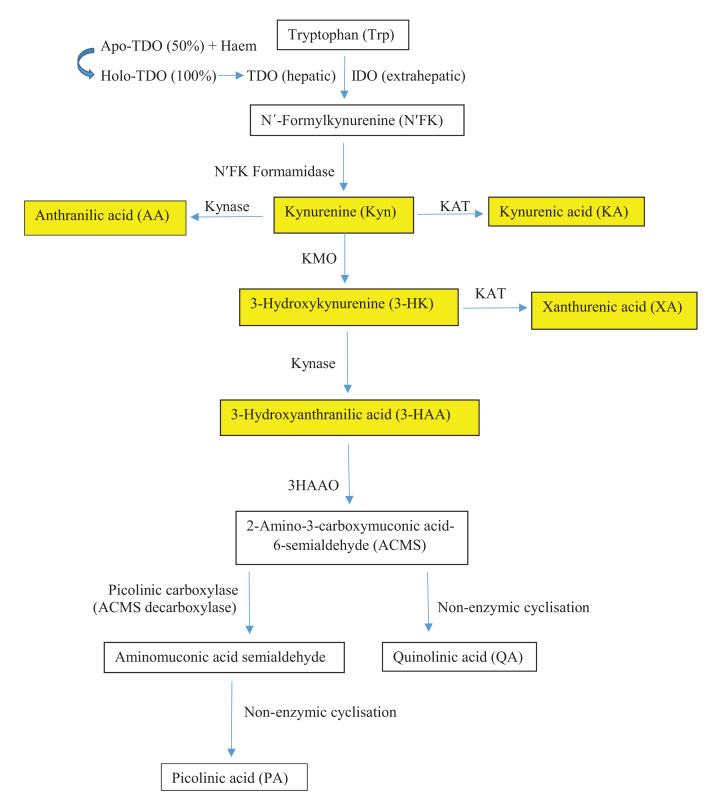
The KP of tryptophan metabolism up to the quinolinic acid and picolinic acid steps Adapted here from Figure 1 in [42] [Badawy, A.A.-B. (2017) Kynurenine pathway of tryptophan metabolism: regulatory and functional aspects. *Int. J. Tryptophan Res.***10**: 1–20. https://doi.org/10.1177/1178646917691938]. Metabolites highlighted in yellow are reactants and products of PLP-dependent enzymes.

The above two studies [1,2] demonstrated that circulating levels of folate and B_12_ were not impaired in AIP, whereas those of B_6_ (expressed as PLP) were. Although plasma [PLP] is the most commonly used marker of the vitamin B_6_ status, it can be influenced by factors such as carbohydrate intake (causing a decrease), fasting (an increase), low albumin levels in critical patients and late pregnancy (a decrease), changes in non-specific alkaline phosphatase, as in hypophosphatasia (an increase) or rickets (a decrease), inflammation and renal dysfunction (a decrease) (see [35–37] and references cited therein). Erythrocyte PLP is a more sensitive marker of the B_6_ status and is not influenced by inflammation or changes in alkaline phosphatase [35]. However, [PLP] is likely to be much higher in human liver than in plasma. For example, in rats, liver [PLP] is considerably higher than that in plasma (32–40 vs 0.4–0.7 µM), with human plasma containing ∼27–202 nM [36,38–41]. Also, the effect of B_6_ deficiency on liver [PLP] is much greater than that on plasma [PLP] (56 vs 9% decrease) [40], thus further suggesting that a functional B_6_ deficiency and/or its extent in liver may not be detectable simply by measuring plasma [PLP]. From the above account, it may be concluded that assessment of the B_6_ status should not rely solely on determination of plasma or erythrocyte [PLP], but should include additional measures. The liver is a major source of PLP formation and for the human liver PLP status that is most relevant to the present discussion, and in the absence of a justifiable liver biopsy, indirect methods should be used. The hepatic functional B_6_ status can be assessed indirectly by two tests based on the amino acids Met and Trp. The Met loading test (leading to elevated levels of urinary cystathionine in B_6_ deficiency) is, however, difficult to interpret under conditions of PLP deficiency, as the test does not currently have the means of distinguishing between impaired activity of CBS and also that of the cystathionase activity of CγL, both of which utilise PLP as cofactor (see below). A more clear-cut measure is that of assessment of PLP-dependent Trp metabolites of the KP (Figure 3).

## The KP of tryptophan metabolism as a tool for assessment of the hepatic functional B_6_ status

### The KP

The KP is the major tryptophan-degradative pathway, accounting for ∼95% of dietary Trp metabolism, with the hepatic pathway handling ∼90% and other tissues including immune cells handling the remaining 5% [42]. The pathway is controlled mainly by the first enzyme: TDO in liver and indoleamine 2,3-dioxygenase (IDO) elsewhere. PLP-dependent enzymes of the pathway are KAT and Kynase. Both enzymes act on two substrates each and both are assigned here as A and B, though KAT exists in four isoforms designated I, II, III and IV (see below). In the present discussion, KAT A converts Kyn into KA, whereas KAT B converts 3-hydroxykynurenine (3-HK) into XA. Kynase A converts Kyn into anthranilic acid (AA), whereas kynase B converts 3-HK into 3-hydroxyanthranilic acid (3-HAA). Both KAT and Kynase in human liver exhibit different substrate preferences and affinities (see below).

In liver of humans, rats, mice and certain other species, TDO exists in both the haem-free inactive apoenzyme and haem-containing active holoenzyme in roughly equal proportions, such that saturation of the enzyme with haem is ∼50%, defined as the percentage haem saturation (100× holoenzyme activity/total enzyme activity) or ∼1, defined as the haem-saturation ratio (holoenzyme activity/apoenzyme activity). Certain other species (e.g. guinea pig, golden hamster, ox, sheep and rabbit) do not possess the free apoenzyme and their TDO therefore exists only as the active holoenzyme that cannot be activated by the addition of haem. Unlike the TDO of rats, mice and humans, the TDO of these other species does not respond to glucocorticoid induction, but can process Trp relatively rapidly, as they are sensitive to Trp toxicity (see [42] for discussion and references). As will be discussed below, these species differences may provide opportunities of assessing the role of haem in CBS activity. IDO also exists fully saturated with haem.

### Testing the functional capacity of the KP by measuring urinary metabolites

This was developed largely by the group of the late R.R. Brown at Wisconsin, U.S.A., and involved measuring the urinary excretion of the various pathway metabolites before and after an oral Trp load, usually of 2 g [43]. Increases in urinary Trp metabolites of the KP occur after Trp loading, with little differences between males and females [43], but with very high levels, particularly in females, if a larger Trp dose (100 mg/kg body weight) is used [44]. The disadvantages of Trp loading doses > 2 g are TDO activation, greater elevation of Kyn metabolite levels, and modulation of pathway enzyme activities by the combined rise in metabolic intermediate levels. In normal subjects, levels of various Kyn metabolites are elevated in 24 h urine specimens by 35–329% after a 2g Trp load ([43,45], see also [46]). The elevation of concentrations of Kyn, KA, 3-HK and XA in males is 2.63-, 4.29-, 1.95- and 2.91-fold respectively [43], with little difference from females (2.81-, 4.49-, 2.01- and 3.68-fold). However, with B_6_ deficiency induced by deoxypyridoxine or isoniazid, huge elevations in levels of these urinary Kyn metabolites have been reported in graph forms, with levels of 3-HK and Kyn accounting for 10–25% and 5–10% respectively of the 2 g Trp loading dose [47]. A representative example of increases in Kyn, 3-HK and XA in individual patients receiving deoxypyridoxine or isoniazid illustrates these huge increases: 17.7-, 26.0- and 18.2-fold respectively, whereas [KA] was increased only by 2.14-fold by deoxypyridoxine, but decreased by 70% by isoniazid [48]. In general, [KA] remains unchanged or is decreased in B_6_ deficiency (see below).

### Urinary XA: the primary marker of B_6_ deficiency after acute tryptophan loading

In normal subjects in the absence of confounders, urinary [XA] is the major index of the B_6_ status, with values <65 µmol/day being considered indicative of adequate pyridoxine intake [41]. The reason why XA is a good indicator of the B_6_ status is that, in B_6_ deficiency, XA excretion is greatly enhanced, in preference to that of the other transamination product KA. Thus, B_6_ deficiency in swine increases the urinary excretion of XA, but not that of KA, despite the simultaneous and considerable Kyn elevation [49], with similar findings having been reported in rats, dogs and humans (see [43] and references cited therein). A potential explanation of the XA elevation in B_6_ deficiency is the profound (62-fold) increase in urinary levels of its 3-HK precursor following oral Trp loading (from 40 to 2500 µmol/24 h) [47]. Assuming a 0.8–2 l volume of a 24-h urine collection, the [3-HK] under these conditions can vary between 1.25 and 3.12 mM.

[3-HK] can be increased by enhanced formation from Kyn by Kyn monooxygenase, decreased hydrolysis by kynase or decreased transamination by KAT. In B_6_-deficient rats, Kyn monooxygenase activity is enhanced, but KAT B inhibition can be ruled out in view of the great elevation of [XA] [50]. Kynase may also be inhibited in B_6_ deficiency by 3-HAA, as has been shown in livers of rats [51] and pigs [52]. Kynase inhibition by 3-HAA in pig liver was 53% at 100 µM and 79% at 1 mM. In humans, there is a similar, but indirect, evidence of Kynase inhibition by 3-HAA, whose urinary levels are also increased along with [3-HK] [53,54], thus suggesting a product inhibition. In pig liver, the Kynase inhibition by 3-HAA is uncompetitive with the 3-HK substrate [52].

### Absence of elevation of urinary [KA] in B_6_ deficiency

The absence of an increase in urinary [KA] in B_6_ deficiency, despite the increase in [Kyn] may be explained by a KAT inhibition. KAT can be inhibited not only by a PLP deficiency, but by other means. As stated above, KAT exists in four isoforms. KAT I and KAT II (acting on Kyn) are highly expressed in liver and deletion of the KAT II gene results in dramatic decreases (>90%) in hepatic KAT activity and [KA] in mice across ages [55]. Lesser decreases in these two parameters (40–50%) are observed in brain under the same conditions and only in young mice (14–28 days old) [55]. In rat liver, KAT II exhibits a ten-fold greater preference for 3-HK, than for Kyn [56]. In mice, KAT III (possibly = KAT I), which is also specific for KA production from Kyn, is inhibited by 3-HK [57]. Other inhibitors of KAT I of relevance to the present discussion are cysteine, Trp and its transamination metabolite indol-3-ylpyruvic acid (IPA) [58]. Additionally, Trp and the IPA metabolites indole-3-ylpropionate and indol-3-yllactate are specific KAT I inhibitors [59]. While inhibition of KAT activities by these various agents *in vitro* is achieved by relatively high concentrations (>100 µM), though with Kyn as substrate at also high concentrations (2–5 mM), it is possible that relevant inhibitory levels could be achieved *in vivo*, especially after acute Trp loading, with which the B_6_ deficiency is revealed. KAT IV is synonymous with aspartate aminotransferase and its use as a measure of B_6_ deficiency is not recommended as its activity can be influenced by liver dysfunction and other clinical conditions. Future mechanistic studies with nutritional and drug-induced B_6_ deficiencies are likely to be more informative of the KAT status.

### Plasma kynurenine metabolite concentrations and ratios as measures of the hepatic B_6_ status in humans

With the advent of more sensitive analytical procedures for measuring low levels of Kyn metabolites, plasma and serum became the major sources for assessing the B_6_ status. The advantages of plasma over urine is that metabolite levels in the latter reflect total body metabolism. In particular, the kidney plays an important role in Kyn handling and disposition [60], and is the second richest source of KAT I after the liver and the richest in glutamine aminotransferase activity [61]. Ulvic et al. [62] proposed the use of substrate/product ratios as measures of the B_6_ status and reported strong negative correlations between the [3-HK]/[XA], [3-HK]/[3-HAA] and [3-HK]/[KA] ratios and decreasing B_6_ levels and that the first of these ratios shows the strongest correlation with PLP levels. A mathematical model based on literature data [63] predicted, among others, increased levels of Kyn, 3-HK and XA and decreased levels of AA and KA in B_6_ deficiency.

Kyn metabolite ratios have also been expressed by this author and many others as product/substrate ratios, with the [Kyn]/[Trp] ratio having been used for many years as an indirect measure of IDO activity, though also of TDO activity and other determinants of Kyn metabolism [64]. In a study in rats treated chronically with the aromatic *L*-amino acid decarboxylase inhibitor benserazide, which also inhibits both KAT and Kynase activities by inactivating PLP by virtue of its hydrazine structure, we [65] observed decreases in the ratios in liver of [AA]/[Kyn] (Kynase A) of 47% and of [3-HAA]/[3-HK] (Kynase B) of 89%. With KAT, the [KA]/[Kyn] ratio (KAT A) was not altered, whereas the [XA]/[3-HK] ratio (KAT B) was decreased by 96%. By contrast, the changes in ratios in serum did not reflect the hepatic changes in all four expressions. The ratios in serum for KAT A and Kynase B were not altered by benserazide: only those for KAT B and Kynase A were decreased by 52 and 78% respectively. This suggests that circulating levels of Kyn metabolites and their ratios do not always reflect those in liver, presumably because of additional contributions from extrahepatic Trp and Kyn metabolism. Acutely, benserazide decreases liver AA, 3-HAA, KA and Trp, but increases Kyn and 3-HK, thus suggesting inhibition of KAT A and Kynase A and B [65].

### The role of tryptophan loading in revealing a functional B_6_ deficiency

Trp loading may reveal the functional B_6_ deficiency by a number of mechanisms: (1) producing higher and more consistent levels of urinary Kyn metabolites than is the case under basal conditions, which show daily and individual variations due to nutritional and physiological confounders [66]; (2) easier measurements of the higher levels of Kyn metabolites by the then available analytical techniques; (3) inducing kinetic changes in PLP-dependent reactions rendering them vulnerable to B_6_ deficiency. The Trp dosage determines the response of the KP enzymes, with small doses only undergoing flux down the pathway and higher doses additionally activating TDO. In rats, a 50 mg/kg body weight dose of Trp does not activate TDO [29] and may therefore act mainly via its flux. From detailed Kyn metabolite data obtained over a 4-h time-course study in rats receiving the above dose of Trp [65], calculation of metabolite ratios demonstrated no significant changes in KAT B, Kynase A or Kynase B, with KAT A (Kyn→KA) being enhanced only at 4 h. Thus, whereas Trp administration to rats at this dose level increases liver Kyn metabolite concentrations, it exerts a minimal effect on PLP-dependent enzyme activities as assessed by the above ratios.

Kyn metabolite ratios have also been assessed in human plasma under basal conditions and after Trp loading. In the study by Ulvic et al. [62] in a large Norwegian sample (*n*=2628), product/substrate ratios for KAT A, KAT B, Kynase A and Kynase B obtained from largely non-fasting subjects were calculated by this author as 0.028, 0.49, 0.008 and 1.19 respectively. The corresponding values in our study with 114 (U.S.A.) fasting subjects of various ethnicities [67] were largely confirmatory: 0.036, 0.516, 0.032 and 0.90 respectively. From these baseline data, it appears that, in humans, KAT and Kynase prefer 3-HK to Kyn as substrate. This is further supported for Kynase by the greater affinity of the human liver enzyme towards 3-HK (*K*_m_ = 77 µM) than towards Kyn (*K*_m_ = 1 mM) [68] with a 15:1 ratio of activity toward these two substrates. This selectivity is supported by our study [67] in normal United States’ subjects in which we reported ratios of Kynase B to kynase A (estimated from the fasting plasma [3-HAA]/[3-HK] and [AA]/[Kyn] ratios respectively) consistent with a much greater kynase B activity, with ratios of 20:1 (men, *n*=54), 40:1 (women, *n*=60) and 28:1 (the combined group, *n*=114). The recombinant human Kynase shows a very high specificity for 3-HK (*K*_m_ = 3 µM) and is inhibited by L-Kyn (*K*_i_ = 20 µM) and also by its 3-HK substrate, as suggested by its sigmoidal activity profile [69].

With Trp loading in humans, it is possible to compare expressions of KAT and Kynase activities as a function of the oral Trp dosage from our previous study [67]. Three Trp doses (1.15, 5.15 and 10.3 g), tested over a 7-h time-course period, were administered in an amino acid mixture used generally for assessing the role of serotonin in behavioural and other conditions. Of the amino acids other than Trp present in this mixture, Leu is known to enhance the flux of Trp through, and also to activate, TDO [67]. To minimise these effect of Leu, the 1.15 g Trp dose was given with a low Leu (LL) content (4.05 g in a 51.25 g formulation). Another 1.15 g dose was given with the medium Leu (ML: 6.75 g in a 51.15 g formulation) content traditionally used in this test formulation. The 5.15 g dose was also given with the ML content, whereas the 10.3 g dose was simply double the 5.15 g dose formulation, i.e. with a high Leu (HL: 13.5 g in a 110.3 g formulation) content. The 1.15 g dose with LL did not activate TDO, whereas the same Trp dose with ML did, by 1.35- to 2.12-fold at 4–7 h, as assessed by the [Kyn]/[Trp] ratio [67], though this ratio elevation can be partly attributed to increased flux of Trp through TDO. The 5.15-g dose maximally activated the enzyme by 2.40-fold at 7 h, whereas the 10.3 g dose was no more effective (2.48-fold). Flux, calculated as the increase in the measured total [kynurenines] over baseline 0 h values, showed increases of 5.86-, 9.20- and 15.62-fold by the three doses of Trp, with the sum of the contributions of the first two doses (15.06) approaching that of the 10.3 g dose.

**Figure 4:**
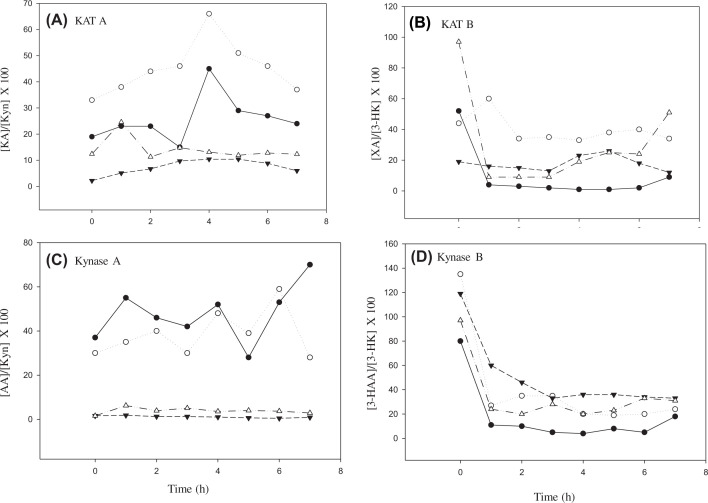
Plasma product/substrate ratios for KAT and kynase after loading with various doses of tryptophan Ratios of: (**A**) KAT A, (**B**) KAT B, (**C**) Kynase A and (**D**) Kynase B were determined before and hourly for 7 h after oral tryptophan loading. Doses of Trp (in g) were 1.15 with low Leu (LL), 1.15 with medium Leu (ML), 5.15 with ML and 10.30 with double the medium Leu (i.e. high Leu: HL). See the text for the leucine contents of these formulations. Symbols: ●Trp 1.15 LL; ○Trp 1.15 ML; ▼Trp 5.15; ML; ∆Trp 10.3HL. Ratios are based on means of these four Trp treatments of normal subjects of *n*=12, 12, 25 and 20 respectively and are derived from data in [67] (Badawy, A.A.-B. and Dougherty, D.M. (2016) Assessment of the human KP: comparisons and clinical implications of ethnic and gender differences in plasma tryptophan, kynurenine metabolites, and enzyme expressions at baseline and after acute tryptophan loading and depletion. *Int. J. Tryptophan Res.***9**, 31–49). For other abbreviations, see the text. The data show that, whereas Kynase A was not altered by any of the doses of Trp relative to zero-time values, KAT A was enhanced by the 1.15 g dose of Trp and only after 3–4 h. By contrast, both KAT B and Kynase B were inhibited by all Trp doses, thus demonstrating the ability of Trp loading to target the 3-HK substrate of KAT B and Kynase B.

The data in Figure 4 illustrate the effects of these Trp doses on KAT and Kynase activities. Whereas Kynase A was not altered by any of the doses of Trp relative to zero-time values, KAT A was enhanced by the 1.15 g dose of Trp and only after 3–4 h. By contrast, both KAT B and Kynase B were inhibited by all Trp doses, thus further suggesting the ability of Trp loading to target the 3-HK substrate of KAT B and Kynase B. From the data in Figure 4, it appears that most of the effects of Trp loading are manifest by 7 h following oral intake. It is therefore of interest that Coursin [70] recommended that, while most Kyn metabolites are recovered in urine within 8 h, the duration of the oral Trp loading test should be standardised by its extension to a 24-h urine collection. The apparent decreases in KAT B and Kynase B suggest that Trp loading inhibits both enzymes, possibly, as discussed above, by Trp, Kyn, 3-HK, IPA and its metabolites (KAT) and by Kyn, 3-HK and 3-HAA (Kynase). These effects of Trp loading mimic those of B_6_ deficiency. The zero-time values in Figure 4 and also those reported by Ulvic et al. [62] further suggest that the basal KAT B and Kynase B activities are much larger than those of KAT A and Kynase A.

In experimental nutritional B_6_ deficiency, kynase activity is impaired, as evidenced by decreased ^14^CO_2_ production from methylene- or uniformly labelled ^14^C-Trp and increased urinary excretion of 3-HK and XA in mice [71] and decreased enzyme activity and increased hepatic [Kyn] and [3-HK] in rats [50]. These changes in B_6_ deficiency compounded by the above effects of Trp loading in humans combine to reveal the robust changes in Kyn metabolism in functional B_6_ deficiency, with accumulation of 3-HK facilitating the KAT B reaction to produce high levels of the major marker XA, especially as the physiological [3-HK] is well below the *K*_m_ of the enzyme (3.8–5.7 mM) [42].

It is opportune to discuss briefly at this point the impact of vitamin B_6_ deficiency on physiology and metabolism. It is important from the outset to emphasise that most of the absorbed dietary B_6_ is taken up by the liver and rapidly phosphorylated there and in other tissues. Pyridoxine phosphate is oxidised to PLP by an oxidase only in liver, kidney and brain [72], which appear to be the richest sources of PLP (in rats) [73]. PLP cannot cross cell membranes, but can be formed in tissues by phosphorylation of pyridoxal taken up from the circulation. PLP is therefore present in various tissues and its levels can be decreased by B_6_ deficiency, as demonstrated in rats treated with semicarbazide [73] or a pyridoxine-free diet [74]. PLP is a cofactor in a wide range of enzymatic processes, including transamination, decarboxylation, racemisation and β- and γ-eliminations and substitutions [75]. B_6_ deficiency can therefore influence many physiological functions, with negative clinical consequences, a discussion of which is outside the scope of the present text, but some of which may impact AIP pathophysiology. Regarding Trp metabolism, B_6_ deficiency may not be limited to the host hepatic KP, but can involve other Trp-degradative pathways in host and by both gut and resident microbiota [76]. For example, by lowering aromatic *L*-amino acid decarboxylase activity in the gut, a B_6_ deficiency can decrease serotonin and melatonin synthesis and undermine their peripheral activity, such as modulation of gastrointestinal and immune function [77–79]. With microbiota, the B_6_ deficiency can decrease production of tryptamine, indole, indol-3-ylpyruvate and their metabolites potentially undermining, among others, their intestinal protection, protective aryl hydrocarbon receptor (AhR) ligand activity and suppression of production of proinflammatory cytokines and chemokines [80–82]. Other than the occurrence of a proinflammatory environment in AIP, it is currently unknown if any of the above effects of PLP deficiency is related to AIP symptoms.

From the above accounts, it would appear that functional B_6_ deficiency can be assessed by measuring fasting plasma or serum 3-HK, XA and 3-HAA concentrations and their ratios [62]. However, there are currently no formal normal values or ranges for these analytical parameters. In patient populations, data should be compared with appropriately matched control groups. The Trp loading test involving 24-h urine collection may have a role in certain settings. Although a blanket 2 g Trp loading dose has been traditionally used, an alternative dosage based on body weight should be more accurate. Allegri et al. [83] suggested a 50 mg/kg body weight dose, corresponding to 3.5 g for a 70 kg adult. However, as TDO is maximally activated by a 5.15g dose, a 30 mg/kg as a more conservative dose (close to the traditional 2 g dose) to avoid a potential TDO activation, was recommended [67].

## Previous studies of tryptophan metabolism in AIP

Price et al. [84] reported that, in the absence of Trp loading, 24 h urinary Kyn was the only Trp metabolite elevated (by 46%) in acute hepatic porphyria, thus suggesting enhancement of TDO activity. Although IDO induction could also elevate [Kyn], this occurs usually after strong immune activation, and the widely used increase in the plasma [Kyn]/[Trp] ratio as a measure of IDO induction is invariably due to a decrease in [Trp], rather than an increase in [Kyn] [64]. However, after Trp loading, urinary excretion of Kyn, KA, 3-HK and XA was greatly elevated in acute AIP patients compared with controls, suggesting not only enhanced transamination of Kyn and 3-HK, but also increased production of Kyn from Trp, almost certainly by TDO activation [84]. The Kyn elevation was, however, huge (14.5-fold), suggesting that in addition to TDO activation renal handling of Kyn may have played a role. In AIP patients in remission, these elevations were no longer observed. Interestingly, pyridoxine supplementation in two acute AIP patients did not correct these elevations and the authors [84] suggested that electrolyte imbalance impairs PLP activity: a notion supported by the observed therapeutic efficacy of metal chelators. It may be relevant that metal chelators such as salicylate and 2,2-bipyridyl inhibit the rat liver TDO haem-containing holoenzyme activity directly *in vitro* [85]. In a more recent study by Gomez-Gomez et al. [86], only urinary Kyn and AA levels were significantly elevated in a second morning sample, further suggesting activation of TDO and kynase A. The AIP patients in this latter study were symptom-free, but exhibited elevated urinary levels of 5-ALA and PBG. Data from this second morning sample were corrected for urine dilution, but not expressed relative to creatinine: a difference [87] that might impact Kyn metabolite levels and may explain the absence of significance in the elevations of KA and XA. In a third study [88], urinary excretion of KA + XA was not significantly altered over the first 4 days of hemin therapy of 12 AIP patients with recurrent attacks. In this latter study, patients were experiencing acute attacks and were maintained on a carbohydrate diet. The glucose element of such diet can inhibit TDO activity [7], a notion supported by the observed trends towards higher Trp, 5-HT and the 5-HT major metabolite 5-hydroxyindoleacetic acid and lower KA + XA in blood, plasma and/or urine before initiation of hemin therapy [88]. Thus, whereas further studies are required to establish the Trp metabolic status in AIP under well-defined conditions, it can be assumed that Kyn and 3-HK transamination may contribute to the functional B_6_ deficiency in AIP and should be explored in future studies of AIP.

## Competition for PLP by B_6_-dependent enzymes of haem, Hcy and tryptophan metabolism

This competition depends on affinity of the enzymes for PLP, extents of their saturation with PLP and factors influencing their activities, e.g. altered synthesis or substrate availability. Affinity for PLP, as expressed by *K*_m_ values, is summarised in Table 1 [89–95]. As shown, of all the enzymes listed, CBS has the lowest affinity and is therefore at a disadvantage when PLP availability is decreased. Extent of saturation of these enzymes can also play a role in PLP utilisation. With 5-ALAS, the enzyme from rat liver or human erythroblasts is at least 80% PLP-saturated [96,97] and its activity can be greatly impaired under severe conditions of B_6_ deficiency, as occurs in pyridoxine-responsive anaemia [97]. By contrast with 5-ALAS, both KAT and Kynase (from rat liver) are only partially saturated with PLP, by 51 and 59% respectively, with nutritional B_6_ deficiency lowering this saturation to 29 and 9.5% respectively [50]. In normal fibroblast lines, CBS is up to 85% saturated with PLP, whereas saturation is decreased in mutant fibroblast lines from homocystinuria patients, especially those unresponsive to B_6_ [92,98].

**Table 1:** PLP *K*_m_ of PLP-dependent enzymes of haem, Hcy and the KP of tryptophan metabolism

Enzyme	PLP K_m_ (µM) and source	Reference
5-Aminolaevulinate synthase 1 (hepatic)	3 (partially purified from rat liver)	[89]
	1–10 (purified from rat liver)	[90]
5-Aminolaevulinate synthase 2 (erythroid)	0.0215 (purified human recombinant wild-type)	[91]
Cystathionine-β-synthase	52–85 (fibroblasts: controls)	[92]
	145–200 (fibroblasts: B_6_-responsive Hcy-uria)	[92]
	990–4000 (fibroblasts: B_6_-unresponsive Hcy-uria)	[92]
KAT (Kyn→KA)	7 (purified from rat kidney)	[93]
(3-HK→XA)	1.7 (purified from rat liver)	[94]
Kynase (Kyn→AA)	8.8 (purified from rat liver)	[93]
(3-HK→3-HAA)	1.8 (purified from pig liver)	[95]

Substrate availability is also important for enzyme activity. With 5-ALAS 1 from rat liver, substrate affinity expressed as the K_m_ is 10 mM for glycine and 70 µM for succinyl Co-A [89]. The corresponding values for the wild-type recombinant human 5-ALAS 2 of erythroid origin are 9.3 mM and 40.7 µM [91]. Other than diversion of glycine through hippurate formation by substrates of glycine acyltransferase (Figure 1) [7], a shortage of succinyl Co-A could impair the enzyme activity. Such a shortage could theoretically occur through inhibition of cystathionine metabolism or a defect in the tricarboxylic acid (Krebs) cycle. With CBS from rat liver, the *K*_m_ value for serine is 4 mM and that for Hcy is 0.8–2.5 mM depending on subunits [99] and the corresponding values for the enzyme purified from human liver are 1.15 and 0.59 mM respectively [100]. With KAT and Kynase from rat liver, the *K*_m_ values of KAT for Kyn and 3-HK are 0.96–4.7 and 3.8–5.7 mM respectively, whereas those of Kynase for Kyn and 3-HK are 1.0 and 0.077 mM respectively [42,68]. As stated above, the greater preference of human Kynase towards 3-HK is reflected in the ratio of plasma [3-HAA]/[3-HK] relative to that of [AA]/[Kyn] being at least 20:1, depending on gender [67]. The smaller contribution of the Kyn→AA reaction to Kynase activity in humans is further suggested by the increase in AA glucuronide excretion following oral Trp loading of AIP patients being the smallest (1.5-fold), despite the huge (14.5-fold) increase in [Kyn] [84]. As stated above, this elevation in urinary [Kyn] suggests that TDO activity is greatly enhanced in active AIP.

With physiological plasma concentrations of glycine (100–300 µM), serine (66–290 µM) derived from various clinical sources, and of Kyn (2.15 µM) and 3-HK (0.31 µM) (means of *n*=114) [42], and given the above *K*_m_ values, all four enzymes in Table 1 exist only partially saturated with their substrates, with KAT and Kynase being the least saturated. Whereas levels of glycine or serine are unlikely to be decreased in AIP, those of Kyn and 3-HK could be increased if TDO is activated, as suggested from the urinary data described above [84,86]. The stimulus for the KAT and Kynase reactions is therefore substrate availability and this coupled with their high affinity for PLP can deprive CBS from its PLP cofactor. With a physiological adult plasma PLP concentration in the 47–97 nM range [101] or up to 202 nM (see above), competition is likely to be strong, with 5-ALAS 1, KAT and Kynase gaining priority over CBS.

As well as CBS, cystathionine β- or γ-lyase (CβL and CγL) also utilise PLP as cofactor, a decrease in their activities caused by B_6_ deficiency could have a direct bearing on Hcy levels, and there is also evidence from studies on cardiomyocytes that Hcy acting through CBS and H_2_S exerts feedback control of CγL [102] (see the further discussion below).

## CBS, haem synthesis and degradation, and the immune system

### CBS inhibition, haem metabolism and inflammation

The potential inhibition of CBS activity leading to the Hcy elevation in AIP can also have a significant impact on haem synthesis and degradation and immune function, all of which could contribute to AIP pathophysiology. Thus, CBS gene deletion decreases bone marrow expression of 5-ALAS 2 and ferrochelatase and increases blood levels of Fe and interleukin IL-6 [103]. Should potential decreases in ALAS 1 and ferrochelatase also occur in livers of CBS-deficient AIP patients, inhibition of haem synthesis can be expected. Proinflammatory cytokines other than IL-6, namely IL-1α, IL-1β and TNF-α, are increased in a mouse model of homocystinuria and a range proinflammatory cytokines and chemokines [IL-1α, IL-6, TNF-α, IL-17, IL-12 (p70), MIP-1α and MIP-1β] are also elevated in homocystinuria patients [104]. Ischaemia–reperfusion of kidney, which is a rich source of CBS, decreases CBS mRNA and protein expression, thus causing increased proinflammatory cytokine levels [105]. The authors suggested the following mechanism. Ischaemia–reperfusion lowers CBS and also CγL, thereby increasing levels of Hcy and decreasing those of H_2_S and reduced glutathione (GSH) resulting in a proinflammatory response (see also below). Mice lacking the H_2_S-forming CγL also showed decreased CBS expression [106]. By contrast, there is also evidence, as stated above, that CBS exerts feedback control on CγL, as a decrease in the former causes increased expression of the latter enzyme [102]. Symptomatic AIP patients exhibit increased plasma levels of a wide range of cytokines, chemokines and growth factors [107]. These latter authors suggested that inflammation in AIP derives from porphyrin precursors inducing liver damage and decreased insulin release causing 5-ALAS 1 induction. Whether decreased CBS activity is a contributor to inflammation in AIP requires assessment.

The increased formation of IL-6 resulting from CBS deficiency can also induce or potentiate the 5-ALAS 1 induction in liver, as has been demonstrated in human HepG2 cells [108]. In this latter study, IL-6 induced 5-ALAS 1 activity moderately (by 40%), but potentiated by 43% that by dimethyl sulphoxide, a solvent compound that induces an acute phase response [108]. Thus, IL-6 exerts a permissive effect on 5-ALAS 1 induction akin to that of cortisol in adrenalectomised rats treated with 2-allyl-2-isopropylacetamide [109]. The increased circulating levels of some proinflammatory cytokines in symptomatic AIP, notably IFN-γ, IL-1β, IL-6 and TNF-α [107], could induce the extrahepatic enzyme IDO to increase production of Kyn, and hence of KA and possibly also AA, thus increasing the demand for PLP, and also production of proinflammatory Kyn metabolites, notably 3-HK, 3-HAA and quinolinic acid (QA). It is generally thought that the balance between the excitotoxic QA and the cytoprotective KA determines the level of neuronal excitability [110]. Given the decreased formation of KA in functional B_6_ deficiency, it is likely that this balance will shift in favour of QA in AIP. The role of 5-ALA in neuronal dysfunction in AIP may involve these Kyn metabolites with actions at the *N*-methyl-D-aspartate (NMDA) type of glutamate receptors being a common feature (see the discussion in [7,8]). Involvement of these Kyn metabolites in neuronal dysfunction in AIP is therefore a possibility worthy of investigation.

### Further implications of CBS inhibition and plasma Hcy elevation in AIP

In relation to KA, a number of cysteine derivatives, including Hcy, decrease its production in rat cortical slices by inhibiting KAT activity and are considered as endogenous modulators of brain KA formation [111,112]. Hcy exerts a biphasic effect on KA production: enhancement by concentrations of 40–100 µM and inhibition at ≥400 µM [113,114]. [Hcy] of 400 µM are unlikely to be achieved *in vivo* except under certain extreme cases (see below) and it is therefore more likely that KA production will be enhanced by lower levels. Although KA is neuroprotective and possesses antiinflammatory properties (see [115] for a discussion), it also exerts proinflammatory effects and has therefore been described as Janus-faced [116]. KA is the KP metabolite with the highest affinity for the AhR, a ligand-activated transcription factor that can elicit protective and destructive effects on immune function. Elevation of [KA] resulting from that of [Kyn] in AIP [107] may activate the AhR to induce poly (ADP-ribose) polymerase 1 (PARP 1) to precipitate an NAD^+^ depletion [117] resulting in cell dysfunction.

Inhibition of CBS and potentially also of CγL by functional B_6_ deficiency in AIP is likely to impair production of H_2_S and glutathione (Figure 2). H_2_S production is PLP-dependent [118] and is achieved by a range of reactions using Hcy, cystathionine or cysteine [119], with the β elimination in the CBS-catalysed reaction between Hcy and cysteine being the predominant source of H_2_S synthesis [120]. As stated above, both Hcy and H_2_S exert dose-dependent regulatory effects on CBS and CγL in cardiomyocytes, with Hcy down-regulating CBS, but up-regulating CγL, and H_2_S exerting the reverse effects [102]. H_2_S is a modulator of inflammation. It exerts protective effects against Hcy-induced cell damage in brain, heart and kidney [121–123]. In macrophages, Hcy appears to trigger a proinflammatory response by inhibiting CγL-H_2_S signaling by DNA hypermethylation of the CγL promoter [124]. As well as decreased H_2_S production, a B_6_ deficiency in AIP can also impair cysteine biosynthesis from cystathionine, leading to decreased glutathione production and weakened defences against oxidative stress. Under such conditions, Hcy can exert toxic effects that are reversible after restoration of cysteine levels [125]. The importance of CγL in glutathione synthesis is further emphasised by the observed decrease in hepatic and renal glutathione levels in CγL^−/−^ mice [125]. As was the case with CBS variants in homocystinuria patients [98], variants in human CγL show loss of affinity for PLP [126].

Haem oxygenase is a stress response protein with antiinflammatory and cytoprotective properties [127]. An additional effect of Hcy is that of potential inhibition of haem oxygenase in AIP. In mice fed Met to elevate [Hcy] *in vivo* and in HepG2 cells stimulated with added Hcy, mRNA expression and protein levels of HO 1 and superoxide dismutase activity are decreased and oxidative stress markers are increased [128]. Thus, the Hcy elevation in AIP could contribute further to oxidative stress and loss of CBS activity.

The above accounts illustrate the complex nature and consequences of Hcy elevation, including that in AIP, and emphasise the need to dissect the roles of the various changes and interactions in the pathophysiology of AIP.

## Latest studies of and further comments on the Hcy status in AIP

In addition to the study in 2020 by Ventura et al. [2] and that by To-Figueras et al. 10 years earlier [1], further reports of the Hcy status in AIP have since appeared. Both the French and Italian Porphyria Centres and others in the Envision givosiran trial have observed strong elevations of [Hcy] in porphyric patients (see [129] and references cited therein). Notable among these reports are those by Petrides et al. [129] and To-Figueras et al. [130]. In [129], 2 AIP patients receiving 5-ALAS 1 gene silencing therapy with givosiran developed strong elevations of plasma [Hcy] of 100–200 and 100–400 µM. Both patients exhibited strong adverse reactions: a severe systemic allergic reaction in one and fulminant pancreatitis in the other, which could be attributed to the severe Hcy elevation. The authors [129] attributed the Hcy elevations in part to a single mutation in the MTHFR gene and to a givosiran-induced decrease in haem availability. This latter explanation is strongly supported by their observation of a prompt decline in plasma [Hcy] upon haem arginate administration that gave way shortly (3 days) after termination of haem therapy to a return of the elevation induced by givosiran. The study by To-Figueras et al. [130] confirmed the fluctuating moderate elevation of plasma [Hcy] in AIP patients under basal conditions and the greater elevation induced by givosiran therapy, with levels of up to 212 µM having been observed. The latter authors also reported that patients with recurrent attacks requiring haem therapy presented with raised Hcy levels over a long period of observation spanning several years. It is currently unclear if Hcy elevation in AIP patients in the absence of givosiran or other therapies exerts any significant harm, if at all. Yet, potential consequences of a strong Hcy elevation need to be considered and should not be ignored.

Whereas a decrease in plasma [Hcy] by haem therapy is transient, it cannot be reconciled with the ability of haem to cause a CO-induced CBS inhibition. Perhaps, the normalisation of plasma [Hcy] by haem therapy reflects opposite actions by haem on CBS activity: an increase due to increased haem availability for PLP cofactor activity and a decrease due to TDO activation inducing a functional B_6_ (PLP) deficiency. Of these, the first effect should precede the second. Demonstration of a potential biphasic effect of haem on CBS activity will require closer and detailed monitoring of plasma [Hcy] in patients and/or experimental animals over a 24-h period after a single haem infusion or for a longer duration with repeated dosing. The TDO status may also be subject to a dual effect of haem: initial activation by an increase, followed by subsequent inhibition by a decrease, in haem availability. The plasma [Kyn]/[Trp] ratio, a measure of TDO or IDO activity, was reported [130] for nine AIP patients undergoing givosiran therapy over a 6–16-month period, and found to be unaltered. Whereas no definite conclusions could be made of the observed parameters of this ratio, compared with control values in the literature (see, e.g. [67]), 5/9 AIP patients exhibited raised [Trp] (77–102 µM), 6/9 had raised [Kyn] (2.6–6.6 µM) and 7/9 had a raised ratio (4.56–7.01). These observations suggest that givosiran may modulate Trp metabolism along the KP; a possibility worthy of investigation.

Whereas Hcy elevation and CBS inhibition in conditions associated with nutritional B_6_ deficiency or PLP-responsive CBS variants can be reversed by pyridoxine supplementation, the same may not be true for the functional B_6_ deficiency in AIP, as suggested by the failure of pyridoxine to reverse the increases in urinary Kyn metabolites [84]. It is, therefore, reasonable to suggest that the functional B_6_ deficiency in AIP is based on modulation of Trp metabolism along the KP in a dual manner: an initial TDO activation by haem causing PLP depletion via enhanced KAT and Kynase activities, followed by HO 1 induction by haem, depriving TDO of its haem cofactor, thereby blocking production of Kyn metabolites that is essential for the PLP depletion. It has previously been demonstrated in rats that induction of HO 1 by metal cations or arsenic impairs the saturation of TDO with its haem cofactor leading to decreased enzyme activity (see [7] and references cited therein). Changes in TDO activity and levels of Kyn metabolites are likely to be informative of the B_6_ status and hence of CBS and Hcy levels in AIP. In both cases, TDO utilisation of the (free) regulatory-haem pool in the hepatic cytosol may be important in future studies with givosiran.

That the free regulatory-haem pool is decreased by 5-ALAS 1 deficiency has very recently been demonstrated [131] in mice heterogeneous for 5-ALAS 1^+/−^. Free haem in liver was decreased by 30% in 30-week-old mice. A comparable decreases in HO 1 mRNA expression (36%) was also observed and the authors concluded that both 5-ALAS 1 and HO 1 are closely linked to free haem. Total haem, by contrast, was little altered by 5-ALAS 1 deficiency and this may explain the modest, variable and limited effects of givosiran on *P*-450-dependent drug metabolism [132], as *P*-450 does not utilise the regulatory-haem pool [7]. Earlier, it was reported [133] that 5-ALAS 1 deficiency in aged mice by gene knockout impairs glucose tolerance and is associated with insulin resistance. Whether similar changes will occur with givosiran remains to be assessed in future studies.

The TDO haem saturation in mice given givosiran was reported [134] to be unimpaired at 30%, though normal saturation is usually closer to 50%. However, in this study, givosiran was administered to a mouse model of AIP [135] involving repeated phenobarbital treatment of PBGD gene-deleted mice. 5-ALAS 1 activity is greatly enhanced in this mouse model by the phenobarbital treatment [135]. Phenobarbital influences activity and haem saturation of TDO in various ways [136,137]. Acute intraperitoneal administration of phenobarbital to rats (100 mg/kg body weight) does not alter TDO activity or saturation with haem at 4 or 24 h [136]. Oral administration to fed rats of phenobarbital in drinking water (1 mg/ml: ∼100–125 mg/kg/day) increases the TDO holoenzyme activity by 67% without altering the total activity, thus increasing the TDO haem saturation from 46 to 76% [136]. Longer term chronic treatment of rats with phenobarbital, however, inhibits TDO activity [137]. After the initial (24 h) increase in holoenzyme activity and haem saturation, the holoenzyme returns to basal levels on day 3 and remains unaltered thereafter. By contrast, the total TDO activity is inhibited from day 3 onwards reaching the holoenzyme level and remaining inhibited until at least day 40. Phenobarbital inhibits TDO activity via the allosteric inhibitor NADPH [137]. With apo-TDO inhibition, the use of the % haem saturation is inappropriate. It is therefore likely that the normal haem saturation reported by Yasuda et al. [134] is the result of a potential givosiran-induced decrease on which a phenobarbital-induced real increase or an artefactual one due to apo-TDO inhibition is superimposed. Whether apo-TDO activity was inhibited by phenobarbital has not been reported by Yasuda et al. [134]. Even-so, a normal TDO haem saturation is inconsistent with deficiency of 5-ALAS 1 inhibiting haem biosynthesis and decreasing the regulatory-haem pool. The mouse model used by Yasuda et al. [134] may not be suitable for investigating the potential effects of givosiran on TDO activity or saturation of the apoenzyme with haem.

TDO saturation with haem is directly related to availability of the regulatory-haem pool in the hepatic cytosol [7]. So far, only TDO has been shown to utilise this pool. As CBS also resides in the cytosol, the question arises as to whether it also utilises this pool, even indirectly. As the CBS enzymatic assay does not require addition of haem, it may be concluded that CBS is fully saturated with haem. In fact the human enzyme is nearly fully saturated with haem, at a ∼92% level [138]. As has previously been established for TDO [7], whose apoenzyme half-life is ∼2 h, utilisation of the regulatory-haem pool requires a rapid response to sudden changes in haem availability. Other haemoproteins which do not respond to sudden or rapid changes in haem levels (e.g. after acute administration of 5-ALA) have much longer half-lives, e.g. *P*-450 (7–10 and 24–48 h), catalase (29 h or 2.5–3.6 days), with other cytochromes having even longer half-lives [7]. CBS also has a relatively long half-life of 49h in hepatocytes cultured in the presence of methionine [139]. It is therefore unlikely that CBS utilises the regulatory-haem pool, but can nevertheless respond to changes in haem availability in much the same way as other haemoproteins. A time-course study of changes in CBS activity following administration of 5-ALA, rapidly acting inhibitors of haem biosynthesis or inducers of HO 1 may throw light on haem utilisation by CBS.

## Hypothesis

### Outline of the hypothesis

A hypothesis summarising the above accounts is outlined in Table 2. Elevation of plasma Hcy levels in AIP is due to decreased activity of CBS caused by: (1) defective binding of its PLP cofactor; (2) decreased haem availability; (3) inhibition by carbon monoxide; (4) increased PLP utilisation by enzymes of the KP of tryptophan degradation. Haem is thus the primary determinant of, and plays multiple roles in, the CBS inhibition. Decreased PLP availability to and inhibition of CBS by CO occur when haem levels are increased, whereas decreased PLP binding occurs when haem levels are decreased. The hypothesis predicts that: (1) fluctuations in plasma Hcy levels in AIP patients are caused by dynamic changes in haem levels in response to physiological, nutritional and external stimuli; (2) elevation of plasma Hcy levels should occur when haem levels are increased during haem therapy or decreased by nutritional or pharmacological interventions in, or genetic manipulation of, the biosynthetic pathway. With gene therapies, the Hcy elevation is more likely to occur if inhibition of haem biosynthesis is the primary target. This has now been borne out by the recently reported [129,130] Hcy elevation by givosiran.

**Table 2:** The haem status in AIP and its effects on CBS activity

AIP therapy → →	nil	hemin	glucose/givosiran
	↓	↓	↓
Haem status → →	Variable	High	Low
	↓	↓	↓
	↓	↓	↓
CBS activity → →	variable	low	low
	↓	↓	↓
	↓	↓	↓
Mechanisms → →	1. Fluctuations in haem levels	1. Haem induction of HO 1 2. Increased CO production 3. CBS inhibition by CO 4. Haem activation of liver TDO 5. Increased Kyn production 6. Depletion of PLP by KAT and Kynase activation	1. Inhibition of haem synthesis 2. Decreased haem availability to CBS 3. Impaired PLP binding to CBS 4. decreased cofactor activity 5. CBS and CγL variants may occur 6. Possible degradation of variants

### Testing the hypothesis

I would like to invite the porphyria research community to test the above hypothesis at the preclinical mechanistic and clinical levels along the following suggestions.

#### Experimental studies in rats and guinea pigs

Liver 5-ALAS 1, HO 1, TDO and CBS activities and plasma Trp and Hcy concentrations should be studied in rats after acute and chronic administration of hemin (haematin). Changes induced by hemin should be compared with those of glucose to establish if differences could explain the CBS inhibition. With glucose, HO 1 should not be activated, whereas TDO and 5-ALAS 1 will be inhibited. Studies in guinea pigs could be very informative. This species possesses unique properties impacting the haem-biosynthetic pathway and tryptophan metabolism [140–142]. Its TDO exists only as the haem-containing active holoenzyme and does not respond to activation by 5-ALA nor to induction by glucocorticoids. Fasting does not increase 5-ALAS 1 activity in the guinea pig and 5-ALA or haematin administration will not activate its TDO, but may induce HO 1 activity. Should this be the case, it may be possible to dissociate the effects on CBS activity of CO from those of PLP consumption by kynurenine metabolites. Some species other than the guinea pig, e.g. the golden (Syrian) hamster (*Mesocricetus auratus*) also lack the TDO free apoenzyme and the glucocorticoid induction mechanism [141] and could therefore respond to the above challenges similarly to guinea pigs.

A comparison of the effects of fasting (starvation) for 24–48 h in rats and guinea pigs may also be informative. In rats, starvation for 24 or 48 h enhances 5-ALAS 1 activity and causes glucocorticoid induction of TDO. Neither effect is observed in guinea pigs. Whereas HO 1 activity is also enhanced by starvation of rats, the response of the guinea pig enzyme is unknown. If guinea pig HO 1 does not respond to starvation, it would be possible to establish an approximate measure of extent of CBS inhibition by CO, by comparing CBS activity between these two species. Finally, the role of decreased haem availability in CBS inhibition can be examined in rats treated with inhibitors of haem synthesis or inducers of HO1. With givosiran, potential changes in activities of CBS, HO 1 and TDO and the haem saturation of TDO should be explored in normal rats or mice not subjected to genetic manipulations.

### Human studies in AIP patients

In assessing the various aspects of this hypothesis, it is important to define clearly the clinical status of patients and the experimental conditions. Definitions and conditions include during acute attacks, immediately after their resolution or in longer-term remission. With glucose therapy, measurements should be made before and daily during therapy and 1 and 2 days thereafter. With haem therapy, measurements should be made before and daily after start of haem therapy for the duration of the therapy, 1 day later, and subsequently 1, 2, 3 and 4 weeks following cessation of therapy. With gene therapy, measurements should be made before and weekly following the gene therapy dose. The potential effects of concurrent medication should be considered. Measurements should include the urinary haem precursors 5-ALA and PBG, plasma vitamins B_6_, B_12_, and folate, fasting plasma Trp, Kyn, KA, 3-HK, XA, 3-HAA and QA and fasting plasma Hcy, Met, Ser, Cys and cystathionine, CBS activity in peripheral blood mononuclear cells, CBS and CγL variants in both untreated and genetically-treated patients, especially if B_6_ supplementation is ineffective, and finally a range of pro- and anti-inflammatory cytokine profiles should be obtained in untreated and treated patients and correlated with CBS activity, Hcy levels and therapy outcome.

## Therapies to lower plasma Hcy in AIP

Strategies aimed at lowering plasma Hcy levels in general are largely nutrition-based and include consumption of a low protein diet (to avoid elevation of the methionine precursor of Hcy), intake of vitamins B_6_, B_12_ and folate (to enhance Hcy conversion into methionine and degradation to cystathionine), and betaine (to further enhance Hcy conversion into methionine and to undermine proinflammatory responses). The value of betaine as a nutritional factor of a broad biological activity [143] justifies its use in AIP patients. However, caution should be exercised in therapy with and dosage of betaine in CBS-deficient subjects to ensure that methionine concentration does not rise to levels (>500 µM) that can induce brain oedema [144]. With a normal plasma methionine range of up to 40 µM, increases approaching 500 µM should be investigated and appropriate measures to lower the betaine dose and/or protein intake considered. Of the nine AIP patients receiving givosiran [130], plasma [Met] was elevated in four subjects to values of 82–616 µM. It is also important to remember that betaine will not lower plasma [Hcy] if levels of PLP and folate are already adequate: in this situation, betaine can inhibit Met conversion into Hcy [145], thus further potentiating a potential Met elevation.

A low protein diet is already recommended for AIP patients in return for a high carbohydrate intake. Various studies and clinical trials of the above B vitamins and betaine have been performed in conditions of high plasma Hcy other than AIP [143,145–149]. Because blood B vitamin levels may not reflect the status of their metabolically active forms in liver or elsewhere, it would be prudent not to rely solely on blood levels, but use a biochemical response (lowered Hcy level) as a guide to efficacy of administered B vitamins and betaine. A no or poor response to B_6_ alone or in combination with B_12_ and folate may indicate a PLP-unresponsive CBS (and/or CγL) variant. Efficacy of B vitamin supplementation in lowering [Hcy] in conditions associated with Hcy elevation is determined by the underlying mechanism(s). For example, in renal failure, folate is effective, whereas B_6_ is not [150]. Efficacy of folate can be explained by defective remethylation being the prevailing mechanism [151]. Absence of a CBS response to B_6_ irrespective of the defective remethylation may indicate that other factors are involved. In experimental renal failure in rats, activities of the following KP enzymes are greatly enhanced: liver TDO, liver KAT, kidney Kynase and liver and kidney KMO, leading to several-fold increases in tissue concentrations of Kyn, KA, AA, 3-HK, XA and QA [152]. It is possible that provision of PLP by B_6_ supplementation may further enhance KAT and Kynase activities as long as their substrate availability is maintained through TDO activation or other mechanisms. This further suggests that TDO inhibition may be an effective therapeutic strategy to block PLP utilisation by KP enzymes. Similarly, acute pancreatitis in humans (from which one givosiran-treated patients suffered [129]) is also associated with elevated plasma Hcy levels [153], decreased plasma [Trp] (by 43%) and increased [3-HK] (2.75-fold) [154].

Current therapies that act by inhibiting haem synthesis at the 5-ALAS 1 step, such as glucose, hemin or 5-ALAS 1 gene silencing with givosiran will most likely contribute to the Hcy elevation. As a proinflammatory environment is present in AIP and can be induced by Hcy, the potential usefulness of anti-inflammatory therapy should be explored. That the use of steroidal and non-steroidal antiinflammatory drugs is safe in the acute porphyrias is indicated in many published lists, e.g. that in [155]. Examples are: steroidal (beclomethasone, cortisol, dexamethasone, fluticasone, triamcinolone); non-steroidal (aspirin, diclofenac, ibuprofen, indomethacin). However, as steroidal antiinflammatory compounds induce TDO, their use may aggravate the functional B_6_ deficiency. Non-steroidal antiinflammatory drugs may be more suitable, with TDO inhibition having already been reported with salicylate, the active form of aspirin [85] and diclofenac [156]. TDO2 gene expression in mouse hippocampus is inhibited by ibuprofen in parallel with abolition of impaired memory [157] and a similar effect on liver TDO is likely. TDO inhibition has previously [7] been proposed as a metabolic approach to therapy of AIP based on prevention of TDO utilisation of the regulatory-haem pool, leaving it available for 5-ALAS 1 repression, but also now additionally to prevent potential PLP depletion by activation of PLP-dependent enzymes of the KP.

## Conclusions and comments

Whereas mild elevation of plasma Hcy in AIP may not be associated with significant health consequences, stronger elevations are generally undesirable and justify intervention. The plasma Hcy elevation and its consequences are almost certainly caused by decreased CBS and CγL activities resulting from a functional vitamin B_6_ deficiency underpinned by multiple effects of haem. It is hoped that this account has provided a platform for further investigation of the Hcy status and its actions in AIP and will stimulate researchers to explore the present hypothesis at the basic mechanistic and clinical levels. The present account has also highlighted important issues that could be profitably addressed in future AIP-related studies. These include whether decreased CBS activity contributes to the proinflammatory environment of AIP, do changes in KA and QA and their ratio play a role in the neurological features of acute attacks?, does an increase in KA promote PARP 1 expression by AhR activation to induce NAD^+^ depletion?, is there a potential occurrence of CBS and/or CγL variants in AIP?, does the functional B_6_ deficiency in AIP, particularly after givosiran or hemin therapy, modulate the Trp-metabolic activity in the GIT or resident microbiota to undermine their protective effects?

## Abbreviations

3-HAA: 3-hydroxyanthranilic acid
3-HK: 3-hydroxykynurenine
5-ALA: 5-aminolaevulinic acid
5-ALAS 1: 5-ALA synthase 1
AA: anthranilic acid
AhR: aryl hydrocarbon receptor
AIP: acute intermittent porphyria
CBS: cystathionine β-synthase
CγL: cystathionine γ-lyase
Hcy: homocysteine
HO 1: haem oxygenase 1
IDO: indoleamine 2,3-dioxygenase
KA: kynurenic acid
KAT: kynurenine aminotransferase
KP: kynurenine pathway
Kyn: kynurenine
Kynase: kynureninase
MTHFR: N^5^,N^10^-*methylene-tetrahydrofolate reductase*
PARP 1: poly (ADP-ribose) polymerase 1
PBG: porphobilinogen
PBGD: PBG deaminase
PLP: pyridoxal 5′-phosphate
TDO: tryptophan 2,3-dioxygenase
Trp: tryptophan
XA: xanthurenic acid
